# Built environment for physical activity—An urban barometer, surveillance, and monitoring

**DOI:** 10.1111/obr.12938

**Published:** 2019-11-07

**Authors:** Raji Devarajan, Dorairaj Prabhakaran, Shifalika Goenka

**Affiliations:** ^1^ Department of Physical Activity and Obesity Prevention, Centre for Chronic Disease Control New Delhi India; ^2^ Executive Director, Centre for Chronic Disease Control New Delhi India; ^3^ Centre for Chronic Conditions & Injuries, Public Health Foundation of India Gurugram India; ^4^ Faculty of Epidemiology and Population Health, Department of Non‐Communicable Disease Epidemiology London School of Hygiene and Tropical Medicine London UK; ^5^ Department of Social and Behavioral Sciences Indian Institute of Public Health‐Delhi Gurugram India

**Keywords:** non‐communicable disease prevention, obesity‐prevention, social planning, sustainable development

## Abstract

The Lancet Commission on Obesity (LCO), also known as the “syndemic commission,” states that radical changes are required to harness the common drivers of “obesity, undernutrition, and climate change.” Urban design, land use, and the built environment are few such drivers. Holding individuals responsible for obesity detracts from the obesogenic built environments. Pedestrian priority and dignity, wide pavements with tree canopies, water fountains with potable water, benches for the elderly at regular intervals, access to open‐green spaces within 0.5‐km radius and playgrounds in schools are required. Facilities for physical activity at worksite, prioritization of staircases and ramps in building construction, redistribution of land use, and access to quality, adequate capacity, comfortable, and well‐networked public transport, which are elderly and differently abled sensitive with universal design are some of the interventions that require urgent implementation and monitoring. An urban barometer consisting of valid relevant indicators aligned to the sustainable development goals (SDGs), UN‐Habitat‐3 and healthy cities, should be considered a basic human right and ought to be mounted for purposes of surveillance and monitoring. A “Framework Convention on Built Environment and Physical Activity” needs to be taken up by WHO and the UN for uptake and implementation by member countries.

## 1 INTRODUCTION

The intermeshed trap of obesity, under‐nutrition, and climate change, which constitutes the Global Syndemic according to Swinburn et al, is an existential threat to the future of human and planetary health.^1^ High body mass index (BMI) of the population is already leading to over 472 million deaths and 148 million attributable disability‐adjusted life years (DALYs)^2^ in a background of escalating climate change and global warming. High temperatures and rising sea levels threaten to severely impact health and survival and further increase the gap between have and have‐nots.^3^, ^4^, ^5^, ^6^ The atmospheric heat trapping fossil fuel carbon has never reached such high levels before.^7^ The solutions to harness rising BMI levels need to be aligned to the wider context of mitigating climate change and global warming.^8^ A physical activity and climate augmenting, age and disabled inclusive built environment will have triple duty actions, enhance the population levels of physical activity, mitigate climate change, and improve health, wealth, equity, and dignity.^9^, ^10^, ^11^

Any form of activity is better than no activity. Even light PA despite falling short of minimum recommendations is health promoting.^12^, ^13^ The maximum health gains from physical activity happen when inactive people become active, in other words when they reach 150 minutes of moderate to vigorous physical activity (MVPA) per week. Beyond 150 minutes too, there continues to be a dose‐dependent benefit of physical activity, and the US government has recently revised the guidelines and recommends less sitting time and 150 to 300 minutes of MVPA.^14^ Over and above this, benefits continue to increase even beyond 300 minutes and start to plateau only after 750 minutes of activity per week.^15^, ^16^ A constrained built environment leads to unknowing sedentariness in the elderly with associated frailty and possible major health hazards including morbidity and mortality.^17^, ^18^ Similarly, a pathology only leads to disability when the built environment is a barrier.^19^, ^20^, ^21^ Thus, human rights and sustainable development goals (SDGs) require the built environment to be supportive. Effectively, the entire population stands to gain from a physical activity enhancing built environment including the inactive, active, infants, children, elderly, differently abled, and those with morbidities.^11^, ^14^, ^22^

The WHO global monitoring framework for noncommunicable diseases (NCDs) calls for a 10% reduction in physical inactivity by 2025.^23^ These targets cannot be met with the current sluggish trends.^24^ The Global Action Plan for Physical Activity and Health 2018 to 2030, to make up for the lost time, recommends a 15% reduction in inactivity levels by 2030.^25^ Bold actions towards protecting and creating health and environment‐sensitive built environments with dynamic urban barometers for monitoring and surveillance is the need of the hour. Walking is a basic human right and should be attained by one and all, in all countries. Since people have the right to life, and ability to move safely on one's own feet, included must those be on wheel chairs and with special requirements towards a legal remedy if not available.^26^ Even highways need to provide segregated space for pedestrians.^26^ The law is supposed to protect the liberty rights and welfare of all its citizens. That is part of the moral purpose of state laws (government laws), state machinery, land use laws, and built environment laws. Built environment consists of the physical and man‐made surroundings including buildings, parks, schools, transportation systems, land‐use, and other infrastructure. Exhaustive reviews by Heath et al, Barnett Et al, Smith et al, M, Masoumi et al, McGrath et al, Cerin et al, Salvo et al, the NICE guidelines, etc^9^, ^27^, ^28^, ^29^, ^30^, ^31^, ^32^, ^33^ have already reiterated the important role played by the built environment to promote population physical activity levels. The National Academy of Science engineering and Medicine (NASEM) report also provides recommendations to enhance monitoring and measuring of physical activity for children, health care, workplaces, and community settings.^34^ However, much of the evidence is from a developed country perspective. Many cities, across the world, have already got physical activity promoting urban design, where pedestrian priority and dignity is protected, and adequate capacity well‐networked public transport is integral to their planning, laws, and thought processes. A majority of the world, especially the developing countries, nevertheless lag behind.

This review is not a formal systematic review but has been written after a deep review of the formal literature, grey literature, and recommendations. It provides the developing country climate sensitive multidisciplinary perspective embedded in the existing knowledge of physical activity and built environment. It develops a framework for a dynamic urban barometer with relevant indicators, inclusive of the developing country perspective, which would reflect (directly or as sensitive surrogates) the progress and status of different countries, cities' and towns' built environment, and the related polices. These could act as a global observatory helping government and the UN towards monitoring and surveillance at the city, state, country, and global level and sustainable development. It could also contribute to a framework convention on built environment and physical activity, which would be a major step towards achieving the SDGs, basic attainment of human rights, UN‐habitat‐3, and WHOs initiative for age‐friendly cities.^10^, ^26^, ^35^, ^36^

## 2 RESULTS

Eight (8 Ds) urban planning and transport indicators are recommended to increase active transport, use of public transport, and enhance health. At state and city level planning, the distribution of employment across regions, public transport within walkable distance, density and diversity which supports vitality of public transport and businesses, easy access to destinations like workplace, educational institutes, marketplaces, recreational areas, etc. within 30 minutes travel, and design which promotes walkable, safe and attractive catchment areas are recommended.^37^ These should include well‐networked, comfortable, quality, safe, and adequate capacity public transport (a) facilities for active travel, wide pavements, with benches, water fountains safe and convenient crossings with limited car lanes (b) school play grounds and policies for playing (c) at worksite‐ user‐friendly stair cases, space and time for physical activity, walking paths (d) in developing countries‐ urgent attention to walkability from the public stations/bus stops, metro stops to their worksites or residences, safe useable subways (e) safe access to parks and public transport for women and children (f) wide active transport lanes, wide pavements, trees for shade and pedestrian dignity and prioritization, (g) universal designs across setting, and contextualized comfort (like tree shade to the elderly and differently abled) and (h) from a developed country perspective‐mixed land use and residential density.^9^, ^27^, ^28^, ^31^, ^38^, ^39^, ^40^, ^41^, ^42^, ^43^, ^44^ From a developing country perspective, there are other factors that need attention. Also, there is already an unsustainable high density and mixed land use that needs decongestion for health because of specific reasons described in the relevant section.

### 2.1 Mitigating high heat in daily living

Global warming is impacting the entire world with Europe too facing an unprecedented heat wave.^45^ Most of the developing countries face scorching high heat most of the year around, posing a huge barrier to the pedestrians, elderly, and differently abled. The temperatures could vary from 34 to 47°C for majority of the months in a year with consequential high heat‐related morbidity.^3^, ^46^, ^47^ In the coming years, rising temperatures could further negatively impact mobility, health, and quality of life.^48^, ^49^, ^50^ Mortality rose by 2% for every degree rise beyond 36.2°C, and the effect was greater when minimum temperature were greater than 26.5°C.^4^ An initiative to improve extreme summer heat by having tree canopies covered across the city could serve as an example for many others to follow.^50^ This was “once upon a time”, a time tested model in many of the planned cities in developing countries, which has been or is being discarded. A study from India reported lowering of the air pollution and lower temperatures (>5°C) in the street segments with lush green tree canopies on either side of the road. On the other hand, the streets without tree canopies were found to have higher levels of “suspended particulate matter” (SPM) on roads than those with the trees.^51^ Tree shades on concrete pavements had other benefits—they increased the life of the pavements and reduced the pavements' cracking, fatigue, rutting, and shoving.^52^ In essence, high heat which is an important barrier to walking in the developing countries, needs to be countered with lush green, shade‐providing trees (canopies), on either side of the roads/pavements. This will make walking comfortable for all age and people. City planning which is health and climate sensitive along with protection of green spaces, forests and water bodies will thus increase mobility and dignity ofthe pedestrians, elderly, children and the disabled.^9^, ^11^, ^19^, ^26^, ^41^, ^42^

### 2.2 Green spaces: parks and tree canopies

(a) The availability and accessibility of useable, safe, green spaces, and parks within 0.3 to 0.5‐km radius from place of residence (>0.3 ha), which are open to the public. (b) Safe access to the parks and attractiveness of the parks are recommended, as proximity to large green spaces are associated with greater physical activity levels in the population, besides also overall well‐being and health.^38^, ^44^, ^53^, ^54^, ^55^, ^56^, ^57^ On the whole, urban green forests, green parks, tree canopies alongside roads and pavements, green vegetation, and trees alongside buildings contribute through multiple interconnected processes towards promoting physical activity, overall health, and well‐being.^58^ They also lower mortality, act as particle and carbon sinks, thereby reducing the air pollution, lower the scorching heat, mitigate climate change, and lower the greenhouse gas emissions.^59^ Shaded green surroundings also help in lowering the surface and the ambient air temperatures and mitigating the noise pollution. Various studies have investigated the health benefits of greenery near residences, worksites, and educational institutions by examining the cumulative exposure to greenery. All‐cause nonaccidental mortality rates were 12% lower in women living near greener surroundings.^60^ Proximity to greenness/greenery was associated with lower cardiovascular disease and stroke mortality and reduced stress with improved mental health^61^ and overall‐health irrespective of the urbanization and socioe‐conomic status.^62^, ^63^ In addition, it is correlated with better birth weight of babies, lower post‐partum depression during and after pregnancy in mothers, reduction in spectacle use, better cognitive development in children, lower risk of prostate cancer among men, and better mobility and health in the elderly.^62^, ^64^, ^65^, ^66^, ^67^, ^68^, ^69^ Trees/greenery also lower air pollution, both particulate and gaseous‐nitric oxide, enhance property value, reduce fossil fuel consumption due to reduced requirements for air conditioning of cars on the roads and buildings, control water run‐offs and flooding, reduce street repair costs, and provide attractive recreational opportunities for the residents.^52^, ^70^ All the above are aligned to SDGs and UN‐Habitat 3. The heat island effects of urban structures can be addressed with trees and will be aligned to UN‐habitat 3 and SDGs. Sadly, in actual terms, greenery has been found to be lower in the socio‐economically marginalized societies.^71^

### 2.3 Pedestrian priority—pavements, sidewalks, pathways, and safety

Road traffic– and transport‐related injuries are the leading causes of preventable deaths among the youth.^72^ The majority (93%) of these fatalities are reported from LMICs and LICs^73^ and involve pedestrians, cyclists and motorized two‐wheelers.^74^, ^75^ Countries that do not invest in wide pavements, sidewalks, pedestrian priority, and other active transport facilities and safe road designs could eat into 7% to 22% of their per capita GDP growth over a 24‐year period.^76^ Inadequate and inappropriate provision for pedestrians and active transporters in the developing countries leads to compromised safety with consequential decline in its social desirability. This is seen especially in the remodeled cities or newly urbanizing towns in the developing countries. Periodic expanding multilane motor carriageways at the cost of pedestrian and active transport lanes escalates the risk to pedestrians and active transporters even further. Walking and other active travel modalities are a constant struggle, and when the average person is finding it difficult, what would the elderly and those physically challenged must go through. Often a common occurrence are multilane motor carriageways along with narrow or filthy and distressed sidewalks with pedestrians walking on the carriageway/the road itself jostling with the upcoming traffic.^74^ High‐speed traffic alongside sidewalks is a known deterrent to pedestrians.^30^ Frequent maintenance work, road widening, encroachments, garbage dumps, signages, etc make pavements unsuitable for walking. This is common across most developing countries, in the South‐east Asian region and much of Africa. Pedestrians are the most vulnerable among road users.^77^ They may die, while walking for basic facilities, fall in pits and manholes, and get electrocuted in many cities in the developing countries. The worst pavements are visible in the socio‐economically marginalized areas, plummeting safety and inequity further. Safety needs to be a non‐negotiable prerequisite in urban design and road design even before other aspects are looked at.

Pavements, as wide as the roads, making pedestrians feel safe and comfortable, are a critical and basic requirement that needs strong action at the ground level. Walking, pedestrianism, and active travel, with universal design, should be a non‐negotiable component of all planning and transport. Contrastingly, in many European countries and in London, pedestrian priority and pedestrian dignity are upper most in the minds of urban designers and planners. For example, in London, the already narrow roads/motor carriageways with only double carriageways (single on each side) are being narrowed further, to broaden the pavements to more than carriageway widths. Additionally, age and disable sensitivity is integral to their design. It is naturally being done without people having to talk, write, and implore the civic authorities nor is the government flaunting it. This is complemented with a strong underground and overground public transport network.

Historically, some of the world's best pedestrian and physical activity‐friendly cities were laid out many decades ago, not for health concerns but, primarily because of common civic sense for public good.^41^ Such thinking needs to be applauded and restored. Pavements need to be of adequate width, preferably as wide as the motor carriageways in the cities with an unobstructed clean, unencroached walking zone. The kerb height should not exceed 150 mm with kerb ramps disable and age comfortable. Heat mitigations through lush green canopies alongside the pavements and roads, provision of benches, and water fountains intermittently are required especially in the LMICs and LICs. Additionally and most importantly, the accompanying motor carriageway should not be more than two lanes wide. Pedestrians feel threatened with high‐speed multilanes of cars moving along. Urban planning and transport needs to be detailed and pedestrian centric, and age and disable sensitive. For example, the safety of pedestrians and active transporters due to a potential collision between the left‐turning vehicles at intersections and on the crosswalks through the road design needs addressal.^77^, ^78^ Other essentials being‐ frequent zebra crossings/crosswalks withappropriate width and sufficient signal time for pedestrians to conveniently cross‐over.

**Figure 1:**
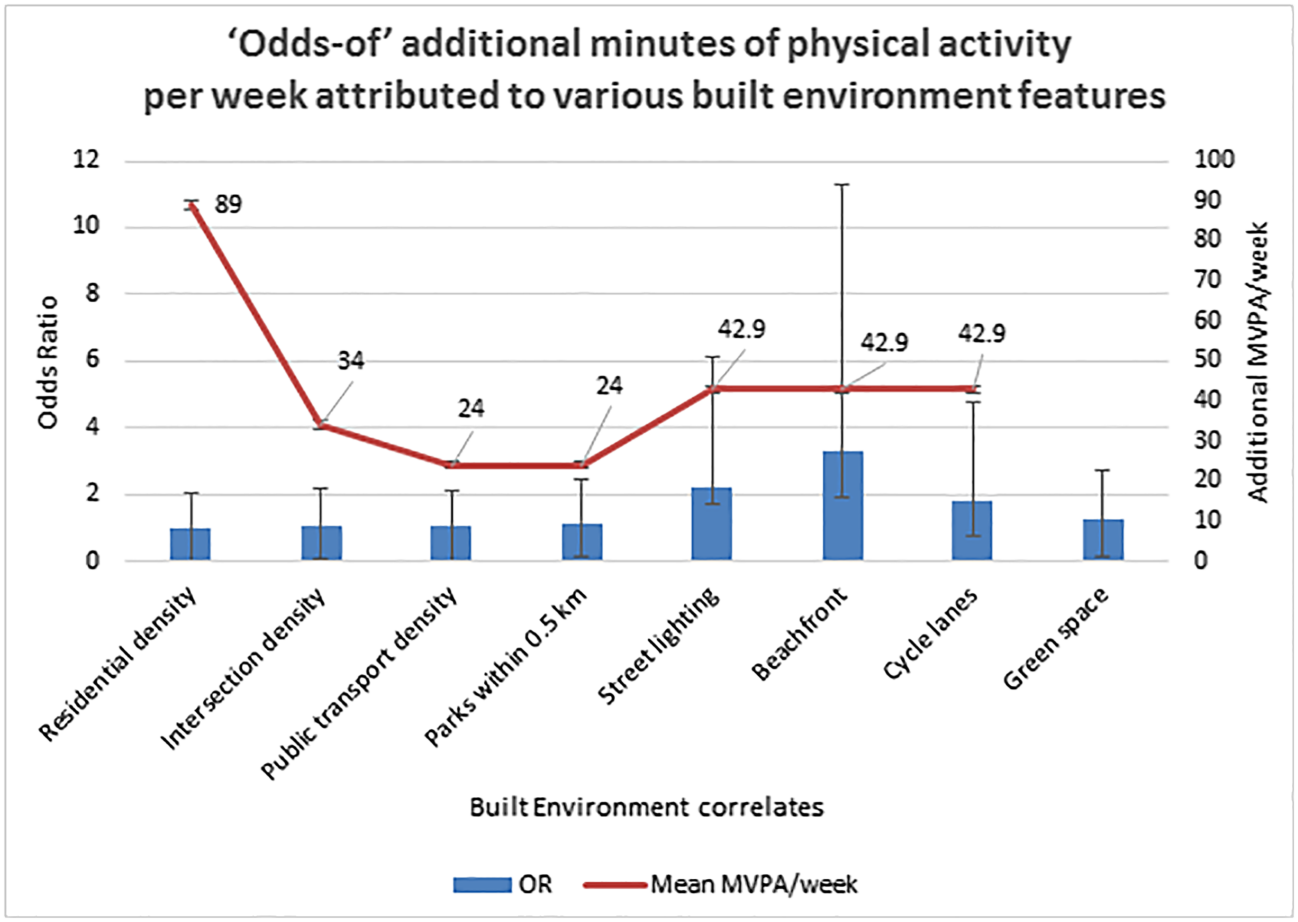
“Odds‐of” additional minutes of physical activity per week attributed to various built environment features. Odds ratio/exp(b)—exponentiation of the coefficients (odds ratio for predictors) and increase in MVPA (moderate to vigorous physical activity) per week are plotted against various built environment features. Sallis et al^38^—Residential density, intersection density, public transport density, parks within 0.5 km. Da Silva et al^79^—Street lighting, beach front, and cycle lanes. James et al^60^—Green spaces

Figure 1 illustrates the established built environment features (*x*‐axis), which enhance the physical activity levels (*y*‐axis), these being, residential density, intersection density, public transport density, parks within 0.5‐km radius, street lighting, beachfront, cycle lanes, and green spaces. In addition, the other attributes, which promoted PA in both adults and children, are a higher proportion of paved streets,^79^ improved neighbourhood walkability, quality of pavements, quality of parks, playgrounds, and their access, and importantly, slower speed of traffic on the roads.^28^, ^80^, ^81^ In developing countries, with 3‐ to 4‐wheeled pushcarts and cycling, the cycling lanes need to be made wider, as wide as the car lanes and provided with lush green tree canopies to protect active travellers from the scorching heat and consequential heat morbidity without the luxury of air conditioning. Green canopies and greenery make walking and cycling attractive, act as carbon sinks, and lower carbon emissions. In many developing countries, highway designs need to consider active transport lanes because people are walking anyway, where they part walk and part hitch rides.

Unlike the developing countries, in developed countries, “walking and walkability” go hand in hand. Therefore, walkability can be assessed by how much people walk. In developing countries, on the other hand, walking and walkability do not go hand in hand as people walk (or cycle) as they have no choice. They walk despite the craters, the non‐useable pavements, high pollution and dust levels, and old motorized transport puffing exhaust black smoke into the noses. They all walk between the upcoming high‐speed highway traffic and parked vehicles—children, those in wheel chairs, and elderly included.^41^ Often, pavements get encroached by either parked vehicles, hawkers, unkempt pavements, construction material, for widening motor carriageways, etc. Other common issues include illegal constructions, hoardings, temporary food stalls, electricity, transformers, garbage, and manholes. Further, poorly enforced rules encourage commercial set‐ups to encroach the footpaths obstructing walkways, which forces pedestrians to use the motor carriageways even with heavy traffic and thereby putting their life in danger. Infrequent or poorly planned zebra crossings further worsens the situation. Extra wide multilane high speed–type motor carriageways are threatening for pedestrians. Adequate lighting on the footpaths and regular police surveillance are other important aspects that have to be ensured for pedestrian's safety. People are jumping over displaced and broken stones, garbage, encroachments dogging motorized two wheelers, and cars to be able to walk. The absence of subways or crossings require pedestrians to dart across, at times, multilane roads, narrowly missing or dodging vehicles, a nightmare for disabled and elderly. Walking and other forms of active transport, thus, get socially downgraded. People give up walking as soon as they can buy any motorized transport—a motorized two‐wheeler or a car or access to any motorized transport—motorcycle, tuk‐tuks, or others. Noise is not even looked at in the overall scheme of things, neither its absence considered a necessity of living nor a consideration in planning. On the other hand, hour‐long, multiple‐lane, traffic jams on many roads is common. In developing countries, there are more than 50 different types of vehicles—both active and motorized. The typical divisions on roads for buses, cars, and cycles will not work in most developing countries.

### 2.4 Equity and pedestrian dignity in developing countries

Pedestrian dignity is paramount for pedestrian activity. Pedestrian dignity in design, which is taken for granted in the developed countries, is lacking in many developing countries. The Scandinavian countries, the United Kingdom, and many European countries are miles ahead of the developing countries when it comes to pedestrian safety, dignity, and comfort; sadly in many cities, in the developing countries across the world, remodelling has seen the pavements literally vanish or become impossible to use. Thankfully, worldwide pedestrian rights are now being looked at as an integral component of human rights,^26^ an important public health priority, and is to be included in the upcoming United Nations SDGs.^35^ Thus land use, urban planning, universal design, which is climate‐resilient from the developing country's perspective needs to be a public health priority.

Providing a pedestrian active transport‐friendly built environment is equity‐promoting.^28^ In LICs/LMICs, on highways where people walk, part walk, and part hitch rides, highway design should consider incorporating active transport facilities for safety, health, and equity. Providing an environment and facilities for sports to deprived and marginalized children is an established instrument for development.^82^

The built environment should directly or indirectly make physical activity in everyday living, the easy choice, the affordable choice, the accessible choice, and the most desirable choice, across the life course, especially in the elderly, the disabled, women, and children.^9^, ^10^, ^11^, ^19^, ^35^, ^36^

How do you action these multidisciplinary complexities at the ground level for population level change? As time is running out, how do you monitor and hold communities and countries responsible and accountable. We developed an urban barometer, which comprehensively captures the wide range of macro and micro structural/environmental social and policy indicators, for active transport, active schools, active cities, and active workplaces, in Table 1. Each of these are aligned to the SDGs. Given the wide‐ranging potential benefits of physical activity promoting built environment for all, we need a Global Framework convention for physical activity, built environment, and city planning. WHO and UN need to table it and motivate countries to be signatories and move forward in a phased manner. A WHO urban barometer observatory would further enhance reaching these goals.

**Table 1:** Physical activity monitoring and accountability indicators

	Individual Indicators—Children and Youth (1)	
Physical activity in children and youth	% of children and youth who meet minimum recommended physical activity guidelines (WHO, American CDC, Australia) both in girls and boys http://www.who.int/dietphysicalactivity/factsheet_young_people/en/ https://www.cdc.gov/physicalactivity/basics/children/index.htm http://www.health.gov.au/internet/main/publishing.nsf/content/health-pubhlth-strateg-phys-act-guidelines#apa512 % of children and youth who indulged in minimum 60 min of moderate‐vigorous aerobic physical activity each day in the last one week and those which include vigorous‐intensity physical activity at least 3 days a week (WHO, CDC, Australia) http://www.who.int/dietphysicalactivity/factsheet_young_people/en/ https://www.cdc.gov/physicalactivity/basics/children/index.htm http://www.health.gov.au/internet/main/publishing.nsf/content/health-pubhlth-strateg-phys-act-guidelines#apa512	SDG 3, 4, 5, 10, 11, 16
Active play	% of children and youth who participate in unorganized/unstructured active play for several hours in a day	SDG 3, 4, 5, 10,
Organized sport/dance participation	% of children and youth who participate in organized/structured active play/dance for an hour or more a day	SDG 3, 4, 5, 10,
Active transportation	% of children (boys and girls) and youth who use active transportation (walking/bicycle) to travel to school, park, market, other out of school activities (developed countries) % of children and youth (girls and boys) who use active transportation to travel to school, park, market, other out of school activities despite have the choice of a car or bus (developing countries) % of differently abled, children, and youth who use active transportation	SDG 3, 5, 11, 13 SDG 3, 11, 13

Environmental Indicators—Children and Youth (2)		
Active transportation	% of children (boys and girls) and youth who use active transportation to travel to school, park, market, other out of school activities % of children and youth who use active transportation to travel to school, park, market, other out of school activities despite have the choice of a car or bus (developing countries)	SDG 3, 5, 11, 13
Safety in active transportation	% of parents who perceive it is safe and feasible to send the children to the local park % of parents who perceive the park is safe to use % of parents who perceive its safe and feasible to send their girls to the local park % of parents who perceive the park is safe to use for their girls	SDG 3, 5, 16
Safety of girls/women	Area wised indicators of girls/women safety in the country	SDG 3, 5, 10, 16
Supportive environment around their home/community as perceived by parents/guardians	Question for parent‐“Please tell me if the following places and things are available to children in your neighbourhood, even if your child [CHILD'S NAME] does not actually use them: 1) park or playground area? 2) a recreation centre, community centre, or boys’ or girls’ court club? 3) sensible safe sidewalks or walking paths to go there” (percentage of parents who answered yes) Same questions in the context of girl child	SDG 3, 5, 16

Environmental Indicators—Active Schools		
Active schools	% of schools which have open play grounds and sporting facilities and equipment and offer them every day to the children	SDG 3, 4, 10
	% of schools which offer PE/dance to >80% children for >150 min per week	SDG 3, 4
	% of schools which have a policy “everyone plays” (including boys and girls)	SDG 3, 4, 5, 10
	% of schools which have a PE period each day (including boys and girls) (from a developing country perspective, it should be divided as) School policies—state, national requirements Proportion of schools required to have dedicated PE period every day. Proportion of school with actual Implementation of PE policies—number of PE hours per week % of schools which are disabled friendly	SDG 3, 4, 5
	% of schools which have a PE period at least 4/5 d a week Or 80% of the working days in a year	SDG 4, 5
	% of schools which actively support active transport to school. “walking school bus,” “walk‐to‐school,” safe routes to school	SDG 3, 11, 13
	% of schools having an after school play/dance program and information for families % of schools having an after school play/dance program and information for families for both sexes	SDG 3, 4 SDG 3,5

Individual Indicators—Adults—Women and Men (3)		
Physical activity in adults (men and women)	% of adults who meet minimum recommended physical activity guidelines % of men and women who meet minimum recommended physical activity guidelines (Men, women) % of adults (men and women) who met the 150 min/week of aerobic exercise guidelines % of adults (men and women) who met the 300 min/week of aerobic exercise guidelines	SDG 3, 5
Active transport to work/college	% of adults who bicycle/walked/used public transport to work/college in the last week % of adults who bicycle/walked/used public transport to work despite having access to cars/private motorized transport in the last week (developing countries) % of women, girls, boys and men who bicycle/walked/used public transport to work/college in the last week % of women, girls, boys, and men who bicycle/walked/used public transport to work despite having access to cars/private motorized transport in the last week (developing countries)	SDG 3, 11, 13, 16 SDG 3, 5
Active transportation for other activities and near home	% of adults who used active transportation to the market, other activities in the last week % of adults who used active transportation to the market, other activities despite having the choice of a car or bus (developing countries) % of women, girls, boys, men who used active transportation to the market, other activities in the last week % of women, girls, boys, men who used active transportation to the market, other activities despite having the choice of a car or bus (developing countries) in the last week	SDG 3, 11, 13, 16 SDG 3, 11, 13, 16 SDG 3 5

Environmental Indicators—Adults (4)		
Community, street scale design, urban planning	% of the population that live within 0.5 km of a park/green space for public use (CDC says 0.5 miles, which is 0.8 km, but recent data from Sallis et al indicate that within 0.5 km as it increases PA in the population. Walkability index of different sections/locations in cities and towns Disable friendliness of the walkability	SDG 3, 11, 13, 15 Data from GIS
PARK—safety and active transportation to the park	% of people who perceive its safe and feasible to go to the nearby park, % of women who perceive its safe and feasible to go to the nearby park, % of people who perceive the sidewalks and crossings are safe to walk to the park (and safety and maintenance of pedestrian paths) % of people who perceive that there are parks useable at a walkable distance from where they live % of disabled who find it safe convenient, feasible to go to the park	SDG 3, 5, 11, 13
Supportive environment—market, college and work	% of people who perceive they can walk to the local market‐ safety, feasibility % of people who feel that they can use active transport and public transport to work/college as its safe and feasible % of men and women, boys and girls who feel that they can use active transport and public transport to work/college as its safe and feasible and the market	SDG 3, 16, 11 SDG 5
Adequate capacity and density of public transports and stops is known to increase physical activity levels	% of people who prefer to take public transport % of people despite having access to personalized cars yet prefer to take public transport % of population who have availability of facilities for recreation, sports, dance within walking distance	SDG 3, 10, 11, 13 SDG 3, 10, 11

Environmental Indicators—Pedestrian Priority, Street Scale Design Street‐Scale and Community‐Scale Design Policy		
	Walkability is how friendly an area is to walking. The international walkability index consists of 4 (1‐4) components or indicator variables (http://health-design.spph.ubc.ca/tools/walkability-index/) of different sections/locations in cities towns: 1. Residential density is the number of houses in an acre of land in a neighbourhood. Higher the value is indicative of more people live in that area. 2. Commercial density is the area meant for commercial use in a neighbourhood. Higher value denotes that there are more businesses, restaurants, retail shops, and commercial establishments in that area. 3. Land use mix is the extent of mixing of residential, commercial, entertainment, office development, etc in a specific area. Higher values denote a balanced distribution of different types of land uses. 4. Street connectivity is the number of street intersections in a neighbourhood. Higher values denote more intersections and better connectivity facilitating easier access between two points. 5. Public transport density (Sallis et al, 2016) 6. Number of useable public parks in the vicinity of 0.5 km (size of the park should be >0.3 ha) 7. Density of bus stations/metro stations/ferry stops Developed country perspective: The above is from a developed country perspective; it is assumed that public transport is safe, and adequate capacity and pedestrian pathways are useable and safe, which may not be the case in a developing country scenario. Developing country perspective: That considered as high density in developed countries is actually low density in developing countries. The walkability may decrease after a certain value high density above in the context of developing countries, where over density is creating a vulnerable unsafe environments to walk and people still walk as there is no other option.	SDG 3 10, 11, 13, 15, 16
	Tackling the contextual barriers to active transport including walkability—developing country perspective: In developing countries, there are many other barriers to walking that include high pollution, high dust levels, and high heat making it uncomfortable, at times prone to dehydration and heat strokes, pollution. There are many more motorized and nonmotorized transports as compared with developed countries. So there is a need for greenery, hydration, segregation of motorized and nonmotorized transports rather than the traditional bus lanes, cycle lanes, and car lanes in developed countries. Plus the population density is many times over.	SDG 3, 10, 11, 13, 15, 16
	Pedestrian paths, street scale design and urban design Pedestrian pathways • % (proportion) of pedestrian pathways, which are safe, unencroached, usability, % of roads—where the width and quality of the pedestrian paths as comparable with the width of road; • % of roads, which have well‐networked pedestrian pathways, crossings, subways • Developing countries: % proportion of interstate highways, which provide for pedestrians and subways as people any ways hitch rides, and there are villages and schools on either side of the interstate highways • Quality of the pedestrian paths—wide, unencroached, well‐maintained—where mothers can walk with child prams, wheel chairs, etc can move. • % of roads in cities/towns/settlements, where there is a definite priority to pedestrians, and nonmotorized transport. • % of roads in cities/towns/settlements where there is a definite priority to disabled and are age‐sensitive for elderly • % of roads, which have green canopies on either side to provide shade (they increase likelihood of active transport and also lower air‐conditioning requirements) • % of roads, where the motorized transport (car, etc) lanes are not more than 10‐11 ft wide, and overall width is comfortable for the Pedestrian • % or roads with “water fountains”—at regular distance/crossings (to counter the hot climate, for hydration purposes) • In tropical countries, high heat countries, green trees for shade—to lower pollution, temperature, comfort in walking and resting on either side of the road. % of roads and pedestrian paths/sidewalks and active transport lane which have shade (cove) provided by lush green trees • % of road length which have benches for people to take breaks and sit. • How you treat your pedestrian_ dignity in being a pedestrian (Respect for pedestrian rights (disabled friendly, universal design, heat mitigation, reinforcement through trees and water fountains). • Adequate frequency of crossings, subways • % of crossings, which have CURB cuts • Car lanes/motorized transport lanes • % of roads where the Width of car lane‐ for car should be less than 11 feet (10‐11 ft), with restriction on number of lanes in cities, so that the road is not too wide and that pedestrians can cross. • Division of road space for motorized and nonmotorized transport • Separation of the motorized and nonmotorized lanes with greenery. Walkability within urban conglomerates/cities, towns Pedestrian priority, safety comfort, need for hydration, greenery, disable friendly. Pedestrian being the heart of city planning Developing countries have more than 50 different types of transport, motorized, and active (nonmotorized). This is unique to developing countries as compared to developed countries of predominantly cars, buses and bicycles. The width has thus to be divided into “motorized lanes(10 ft each approx.), nonmotorized lanes and pedestrian paths	SDG 3, 11, 13, 10
	Active transport (developing countries) Nonmotorized lanes—for multiple different active transport vehicles in developing countries	
	Developing country	
Public transport density	Public transport density—measured on randomly selected routes in each city. Each county and city can decide that.	SDG 3, 10, 11
Priority to other forms of active transport/cycling at crossing	% of crossings in a town/city, where priority is given to cyclists and other forms of nonmotorized transport	SDG 3, 10, 11, 13

Indicators at Worksite		
	1. % worksite/business enterprises, where the staircases/stair wells are well maintained, useable, attractive, and safe, measures that will increase the safety in the using stairs and safety treading, non‐skid tiles or treading, railings, temperature controlled in extreme temperatures. Natural lighting where ever possible. Railing to hold on. Attractiveness of staircases 2. % of worksites encouraging few minutes of physical activity b‐during working hours‐Physical activity breaks 3. Are open spaces available for exercise at worksite or close to worksite, close by parks, walking paths within campuses 4. Are there prompts “point of decision prompts “to encourage people to take the staircase/stairwell 5. In compact spaces, opportunities to be active at worksite 6. % of worksite that have healthy food options 7. % where safe drinking water is freely available 8. % of worksites which are disabled friendly *In developing countries majority of the worksites are unorganized. So most of the above cannot be applied to them	SDG 3, 10, 11, 13
National policies	Cross‐departmental National and state policies Is there an inter‐sectorial, interdepartment platform set up to enhance physical activity in daily living in the population across sectors? Is there a government mandate followed by appropriate action in the different sectors which directly and indirectly promote physical activity Health consideration by ministries of transport, urban development, highways and the environment. Active transport should take priority over other transport by the Ministry of transport and highways and urban development	SDG 3, 4, 5, 10, 11, 13, 15, 16

Abbreviation: SDGs, sustainable development goals.

Sources: Inputs gleaned from various sources, national and regional and global guidelines and contextual from developing countries^9^, ^38^, ^40^, ^83^, ^84^, ^85^, ^86^, ^87^, ^88^, ^89^

Live country‐specific global dashboard will give an opportunity to countries to showcase their achievements and to others to work towards their targets. It can help in surveillance and monitoring. A feedback loop with citizens themselves could further help in implementation.^90^

### 2.5 Public transport increases population physical activity

The use of public transport is an established booster of population physical activity levels. A systematic review by Rissel et al elucidated an increased walking time of 8 to 33 minutes per day in public transport users.^91^ This also saves fossil fuel lowering the carbon emissions. Public transport needs to be age and disabled sensitive, climate sensitive, and should restore the dignity of public transport users in all human habitations.

### 2.6 Net residential density, mixed land use, and destination accessibility

From the developed country perspective, increasing the net residential density, mixed land use, destination accessibility are significantly and positively associated with physical activity.^38^, ^92^, ^93^ High‐residential density, according to the developed country definitions, is known to encourage walking and so do well‐networked streets and close accessibility to shops and utilities. Mixed land development integrates institutional, commercial, and residential uses, thereby providing a purpose to walk. This makes the neighbourhood physical activity–friendly for the pedestrians. Urban sprawls without adequate frequency of mixed land use and crossings discourage physical activity.

In developing countries, on the other hand, the net residential density and mixed land use is already so high that it chokes walkability and basic dignity. People are jostling with cars, buses, trucks, garbage, manholes, hawkers, tricycles, and shop encroachments to be able to walk with safety and dignity severely compromised. People walk as they have no choice and give it up as soon as they can afford their own motorized transport. People live and work in overcrowded localities. The low socio‐economic localities are even more densely crammed where it could go up to 150 000 to 200 000 people per square kilometre. High density, mixed land use, and over‐commercialization give rise to high‐pollution levels, compromised sanitation, and water supply including noise pollution and carcinogenic effluents from small‐scale commercial establishments. This becomes a harbinger of many communicable diseases and NCDs.^94^ For example, a family of 10 packed into 20‐ft^2^ space or 10‐ft^2^ working space, live/work in unhygienic conditions, and compromised safety, with sewerage‐sanitation problems, water shortage, heat‐related morbidity, and infectious diseases in these mixed‐use settlements. So, easing the congestion and decreasing the densities are the requirements, also limiting construction and commercial activity to ensure it is commensurate with the water supply, public transport, road and other civic amnesties with adequate open spaces, and greenery. Also, one needs to be cognizant that in developing countries, where safety is major concern (from crime, rape, and carcinogens) and land mafia encroachments the norm, having recommendations to increase densities and mixed land use will give fodder to legitimize such illegal violations and disease‐creating built environments.

In developed countries however, where there are many kilometres of urban sprawls, cities need to get more compact and move away from being car‐centric environment.^37^ Compact design, with green spaces, improves physical activity levels and health.^93^, ^95^ Residential buildings and educational institutions including schools need to be located away from the high‐traffic zones both in the developing and the developed countries.^41^ Stevenson et al modelled the health benefits to be 420 to 826 DALYs per 100 000 population from compact city scenarios, which included a shift to active transport in Melbourne, Boston, London, Copenhagen, Sao Paulo, and Delhi, except for a small predicted increase in road trauma for pedestrians and cyclists accumulating to 34 to 41 DALYs per 100 000 population.^83^ In contrast to the modelled data by Stevenson et al, analysis of historical data in Denmark overall and in four cities individually, namely, Copenhagen, Aarhus, Odense, and Aalborg, elucidated that there was a 45% decline in injuries related to cycling, despite cycling having gone up by 10% in the period of 1998 to 2015.^96^ In addition, it was estimated that 3328 type 2 diabetes, 5742 CVD, 2076 cancer, and 6190 deaths were prevented because of the increase in cycling. Thus, a well‐designed built environment actually decreased the accidents even as active transport increased. The 8 Ds on urban design and transport interventions are recommended towards making cities compact, which are alluded to in the previous section.^37^, ^38^

### 2.7 Worksite built environment and policies for promoting physical activity

Employed people spend most of their waking time at offices. Worksite‐based programmes provide opportunities for employees to take up physical activity pursuits. These are also known to be productivity and confidence boosters and also reduce absenteeism.^97^, ^98^, ^99^, ^100^ Workplaces surrounded by a variety of attractive and pedestrian‐friendly neighbourhoods encourage walking, bicycling, and use of public transport.^101^ Small changes like introducing a sit‐stand device for the employees could significantly reduce sitting time by 66 minutes per day and achieve health benefits like improvement in neck and back pain.^102^ Staircases as a central and focal aspect of the architecture (as against the elevator) encourages employees to take stairs rather than lifts. Self‐servicing policies for accessing beverages, having lunch in the canteen area rather than at the work stations, 2 minutes standing/walking breaks after every 20 minutes of sedentary work, and availability of yoga and zumba classes before and after work schedules are some of the in‐house interventions that worksites could include in their employee programmes.^103^, ^104^, ^105^ Physical activity breaks for 2 minutes in sitting time every 20 minutes has documented significant benefits.^103^

### 2.8 Schools built environment and policies for promoting physical activity

Children's physical activity should be enhanced through school‐based interventions—a conducive built environment and supportive polices. An exclusive games period of 30 to 40 minutes per day ensures that children engage in WHO recommended MVPA levels of 60 minutes to some extent.^106^ In a recent meta‐analysis by Hollis et al, only 40% of the total school physical education (PE) lesson time was utilized for actual MVPA.^107^ The school‐built environment including availability of facilities and equipment had a great impact in addition.^108^ The school‐built environment has a positive association with MVPA among students (*P* < .001).^109^ A weekly increase of over 4 minutes in MVPA was observed with every unit increase in the built environment score. These units consisted of a gymnasium; a large room for aerobics, zumba, and yoga training; running tracks; outdoor playground; a skating area; indoor tennis, table tennis, and badminton courts; a pool; etc. Delivering physical education instructions for the recommended 30 minutes a day was found to decrease the BMI by 1.56 units among boys.^110^, ^111^, ^112^ Creating a safe and enjoyable environment will encourage girls to engage in physical activity pursuits.^113^ In addition, providing green and safe routes to school will also encourage children and parents to consider walking to school and, thus, increase the physical activity beyond the school premises.^114^

## 3 CONCLUSION

Built environment has a profound influence on the physical activity levels of the population, in preventing a pathology from becoming a disability and enhancing health across the lifespan.^11^, ^19^ Besides, it would have a major role to prevent obesity, lower carbon emissions, and decelerate global warming. In the presence of existing reviews on physical activity and built environment,^22^, ^28^, ^31^, ^39^, ^43^, ^92^, ^115^, ^116^ our review significantly adds to the science in three substantial and different ways. Firstly, it provides the developing country's perspective, which has been missing in all previous such work. The importance of providing the developing country perspective cannot be overstated as 5 of the 7 billion people globally live in the developing countries.^117^ Secondly, our review progresses to an integrated T‐4 translation as it moves to actionable ways to implement the age‐friendly cities of WHO, attainment of WHO goals for prevention of NCDs, SDG goals, 4, 5, 10, 11, 13, 15, 16, UN‐Habitat‐3, and pedestrian, elderly and disable friendliness for human right and science at the broader country and global level through a multisite monitoring and accountability framework. Having already fallen back on the UN targets of a 10% reduction in physical inactivity by 2025, country action at the ground level along with a strong monitoring and accountability framework can help us reach our goals. Thirdly, these interventions that enhance population levels of physical activity can act towards triple duty action for policy makers.

Citizen's health and well‐being needs to be a fundamental right in each country's constitution. This will enable other departments and ministries to look at health and environmental issues for prevention and sustained health. As of now in many developing countries, health is only visited and owned by the Ministry of Health for therapeutic care. Health should be the responsibility of all ministries and departments. Every minister needs to be a Health Minister. Active transport and age‐disabled friendly pedestrianism should be an integral component of planning of all Ministries‐inducing transport. A “Framework convention on physical activity and built environment in daily living” needs to be urgently taken up at the global level for meaningful results towards obesity prevention, climate change mitigation, SDGs, disabled and age inclusiveness, dignity, and prevention of NCDs at the ground level. The monitoring and accountability indicators can be adapted by each country. Pragmatic and sustainable interventions are needed to counter the rapidly growing inactivity, by providing environments that change the way we live in our daily lives. A health‐promoting and sustainable built environment can have a profound influence on the population levels of physical activity, climate and environmental protection, human rights, overall health and well‐being, and equity—both socio‐economic and age/disabled sensitive. Making walking pedestrian priority and other forms of active travel the comfortable, easy, preferred choice, supported by a well‐networked, safe, comfortable, adequate capacity public transport system, access to well‐maintained public parks (>0.3 ha) and green spaces (>0.3 ha) within 0.5‐km radius, green canopies alongside carriageways and pavements, trees along buildings‐constructions, are some important measures. A tropical country's city's handling the onslaught of heat through trees along pavements, large green spaces, quality of public transport, air pollution, noise decibels, pedestrian dignity, dignity for elderly and disabled, active transporter dignity, safety, cap on the maximum density per square kilometre are some of the critical requirements from a developing country perspective.^118^ Adequate and appropriate green cover, green canopies for shade are a dire requirement in addition to safe hydration through potable water and related facilities. Pedestrian, elderly, and disabled dignity and comfort is prioritized in most developed countries and needs urgent action in the developing countries. It is high time that the built environments become creators of health, equity, and environment.

## CONFLICT OF INTEREST

We have no other relevant disclosures.
